# *Bacillus lumedeiriae* sp. nov., a Gram-Positive, Spore-Forming Rod Isolated from a Pharmaceutical Facility Production Environment and Added to the MALDI Biotyper^®^ Database

**DOI:** 10.3390/microorganisms12122507

**Published:** 2024-12-05

**Authors:** Luciana Veloso da Costa, Juliana Nunes Ramos, Leticia de Sousa Albuquerque, Rebeca Vitória da Silva Lage de Miranda, Talita Bernardo Valadão, João Flávio Carneiro Veras, Erica Miranda Damasio Vieira, Stephen Forsythe, Marcelo Luiz Lima Brandão, Verônica Viana Vieira

**Affiliations:** 1 Institute of Technology in Immunobiologicals, Oswaldo Cruz Foundation, Rio de Janeiro 21040-900, Brazil; 2 Oswaldo Cruz Institute, Oswaldo Cruz Foundation, Rio de Janeiro 21040-900, Brazil; 3 Foodmicrobe.com Ltd., Adams Hill, Keyworth, Nottingham NG12 5GY, UK; sforsythe4j@gmail.com

**Keywords:** *Bacillus lumedeiriae*, bacterial identification, pharmaceutical industry, genomic taxonomy, MALDI-TOF MS, 16S rRNA

## Abstract

A Gram-positive, aerobic, rod-shaped and spore-forming bacterium strain designation, B190/17, was isolated from an air monitoring sample of a Brazilian immunobiological production facility in 2017. The strain was not identifiable by biochemical methodology VITEK^®^ 2 or by MALDI-TOF MS with VITEK^®^ MS RUO and MALDI Biotyper^®^. The 16S rRNA gene sequencing results showed 98.51% similarity with *Bacillus wudalianchiensis* FJAT 27215^T^, 98.28% with ‘*Bacillus aerolatus*’ CX 253^T^, 97.96% with *Bacillus badius* MTCC 1458^T^, 97.63% with *Bacillus xiapuensis* FJAT 46582^T^ and 97.21% with *Bacillus thermotolerans* SGZ8^T^. Biochemical data showed that the strain was alanine arylamidase-, Ala-Phe-Pro arylamidase-, ELLMAN (cysteine residues)-, leucine arylamidase-, phenyalanine arylamidase- and tyrosine arylamidase-positive. The genomic DNA G+C% content of B190/17 was 41.6 mol%. The phylogenetic, genomic taxonomy and biochemical tests suggested that B190/17 represents a novel species and should be classified as the type strain of a novel *Bacillus* species. The name *Bacillus lumedeiriae* sp. nov. was proposed. After characterization, B190/17 was added to the MALDI Biotyper^®^ database as *Bacillus lumedeiriae* sp. nov.

## 1. Introduction

Microbial contamination is one of the main problems in the pharmaceutical industry. This is of particular concern during the manufacture of thermosensitive sterile products such as vaccines and other immunobiologicals, as these cannot be subjected to terminal sterilization. Consequently, these products need to be produced aseptically according to strict quality assurance requirements [1,2]. In 2022, the European Medicines Agency established the Contamination Control Strategy (CCS) approach. This aims to identify microorganisms isolated from clean areas and to evaluate the risk of their presence in environmental production. Subsequently, appropriate measures can be put in place to eliminate high-risk contamination and ensure safe and quality products [3]. To achieve this goal, the identification of microorganisms isolated in pharmaceutical production plants is essential [4,5].

Various methodologies can be employed for microbial identification in the pharmaceutical industry, ranging from biochemical methods to genomics. However, these are laborious and expensive to implement [1,2,6]. Although biochemical methods are cheaper and easier to perform than DNA sequence-based methods, their databases are often limited to more frequently isolated species. In addition, microorganisms isolated from nutrient-limited clean production areas which are subject to the frequent use of disinfectants may not express their standard characteristics in biochemical tests, resulting in inaccurate, or even incorrect, results [1,4,7]. Previous studies have shown that matrix-assisted laser desorption ionization–time-of-flight mass spectrometry (MALDI-TOF MS) is a significantly faster and more accurate identification system than conventional biochemical methods. This method is based on the extraction of proteins from whole microbial cells. Although MALDI-TOF MS databases have improved over the years, some environmental species cannot be identified. This limitation applies to *Bacillus* and related genera, which are among the main bacteria isolated in pharmaceutical industries [1,4,7,8]. In these cases, the DNA sequencing of the 16S rRNA gene and other housekeeping genes like *rpoB* (encoding the beta subunit of RNA polymerase) and *gyrB* (encoding the beta subunit of DNA gyrase) can be performed [1,9,10].

Until 2020, the *Bacillus* genus was composed of more than 280 species. However, studies by Gupta et al. [11] and Patel and Gupta [12] demonstrated how taxonomically and phylogenetically varied the genus was. Consequently, they proposed the reclassification of more than a hundred species into new genera [11,12]. Currently, the genus *Bacillus* comprises 111 species with validly published and correct names [13].

Pharmaceutical industry facilities possess potential environments for the discovery of new species of microorganisms which have not been well studied and due to commercial reasons may not have been publicized [1,7,8]. In two previous studies, Costa et al. [1,8] characterized 97 strains of *Bacillus* and related genera isolated from an immunobiological pharmaceutical facility by MALDI-TOF MS and 16S rRNA gene full-length sequencing. These earlier studies include the potential isolation of new bacterial species; however, further investigation was required before their formal recognition could be proposed.

The aims of this study were to achieve the taxonomic characterization of the novel *Bacillus* strain B190/17 and to introduce its spectrum into two MALDI-TOF MS system databases. This strain was firstly analyzed by Costa et al. [8] and was proposed, in this study, as *Bacillus lumedeiriae* sp. nov., with the type strain B190/17^T^ (=CBAS1225^T^ = CCGB2064^T^). In this study, the genome sequence of B190/17 was analyzed to determine the genomic and phenotypic features of the new species.

## 2. Materials and Methods

### 2.1. Bacterial Strains and Culture Conditions

The strain B190/17 was isolated from an air environmental monitoring sample at a Brazilian immunobiological production facility in 2017 [8]. The strain was streaked on Sheep Blood Agar 5% (SBA) plates and incubated at 30–35 °C for 24–48 h. Stock cultures were prepared with Brain Heart Infusion Broth (Merck KGaA, Darmstadt, Germany), containing 20% glycerol (Merck KGaA, Darmstadt, Germany). The strain was deposited at the Coleção de Bactérias do Ambiente e Saúde (CBAS) hosted at Fundação Oswaldo Cruz (Fiocruz), Rio de Janeiro, Brazil. The deposit number was 1225^T^. CBAS is affiliated with the World Federation for Culture Collections (WFCC) and registered as World Data Centre for Microorganisms (WDCM) 958. B190/17 was also deposited at the Coleção de Culturas do Gênero *Bacillus* e Gêneros Correlatos (CCGB) hosted at Fiocruz, Rio de Janeiro, Brazil and the deposit number was 2064^T^. CCGB is affiliated with the WFCC and registered as WDCM 574.

### 2.2. Phenotypic Tests

The B190/17 strain was streaked on Tryptic Soy Agar (TSA) plates (BioCen do Brasil, Campinas, Brasil), incubated at 30–35 °C for 24–48 h and submitted to the commercial test API^®^ 50 CH (bioMérieux, Craponne, France), according to the manufacturer’s guidelines, and to the motility test in Sulphide Indol Motility (SIM) medium (Merck KGaA, Darmstadt, Germany). The strain was also analyzed by the VITEK^®^ 2 Compact System (bioMérieux, Craponne, France) with the BCL card, which was developed for the automated identification of aerobic endospore-forming bacteria. The results obtained by VITEK^®^ 2 with a percentage of probability <85% were considered unidentified. Proteomic analysis with MALDI-TOF MS was performed with two different systems, VITEK^®^ MS RUO (bioMérieux, Craponne, France) and MALDI Biotyper^®^ (Bruker Daltonics, Billerica, MA, USA), according to the manufacturer’s instructions. For both systems, a portion of the colony was applied to the slide in duplicate together with one microlitre of formic acid 70%, and after drying, one microlitre of alpha-cyano-4-hydroxycinnamic acid matrix solution (VITEK MS-CHCA, bioMérieux, Craponne, France and Bruker HCCA, Bruker Corporation, Billerica, MA, USA) was also applied. The calibration of VITEK^®^ MS RUO was performed with *Escherichia coli* ATCC 8739, whereas MALDI Biotyper^®^ was calibrated with the Bacterial Test Standard (BTS) (Bruker Corporation, Billerica, USA). The results were analyzed by SARAMIS Premium software version 4.0.0.14 and MBT Compass HT version 5.1.100. The VITEK^®^ MS RUO and MALDI Biotyper^®^ isolate results with an identity percentage ≤ 75% and scores ≤ 1.70 were considered unidentified, respectively.

### 2.3. Genotypic Identification by 16S rRNA Gene Sequencing

Full 16S rRNA gene sequencing was performed using the MicroSEQ™ Full Gene 16S rDNA kit (Thermo Fisher Scientific, Waltham, MA, USA), according to the manufacturer’s instructions. The prepared plates were analyzed on the 3500 Series Genetic Analyzer (Applied Biosystems, Waltham, MA, USA), and the sequences were assembled using DNA Star LaserGene SeqMan software version 7.0.0. Identification was obtained from the website https://www.ezbiocloud.net/ (accessed on 13 October 2024) [14], and the sequence was deposited in Genbank/NCBI https://www.ncbi.nlm.nih.gov/ (accessed on 14 November 2024) with the access number OK586830.1 [1]. The results retrieved with ≥97.0% identity with EzBioCloud were considered valid for identification at the genus level and ≥98.7% for identification at the species level [15]. The 16S rRNA gene sequences of related taxa were obtained from EzBioCloud and from the GenBank Database https://www.ncbi.nlm.nih.gov/nucleotide (accessed on 13 October 2024). The sequences were aligned with PhyloSuite version 1.2.2 [16,17] with MAFFT version 7 plugin default parameters [18]. A maximum likelihood phylogenetic tree was built using PhyloSuite version 1.2.2 [19] and the IQ-TREE version 2.2.0 plugin [20]. A general time-reversible (GTR) substitution model was used according to ModelFinder plugin. FigTree version 1.4.4 was used to pre-edit the phylogenetic tree, and the final image was obtained with Inkscape version 1.0.1.

### 2.4. Genome Sequencing, Assembly and Annotation

Genomic DNA was extracted and purified using the DNeasy Blood & Tissue Genomic DNA Isolation kit (Qiagen, Hilden, Germany). Next-generation sequencing was performed at Fiocruz Platform Network (Oswaldo Cruz Institute, Rio de Janeiro, Brazil) using an Illumina HiSeq 2500 sequencer (Illumina Inc, San Diego, CA, USA). A library was constructed with the Nextera XT DNA Library Preparation Kit (Illumina Inc, San Diego, CA, USA). The reads were assembled de novo using SPAdes version 3.15.4 [21]. The genomes were annotated using Rapid Annotation using System Technology (RAST) [22].

### 2.5. Phylogenetic Analysis of 16S rRNA, rpoB and gyrB Genes

A concatenated tree was built with the complete sequences of the housekeeping genes: 16S rRNA (previously obtained), *rpoB* and *gyrB* (extracted from the genome with RAST). The 16S rRNA sequences of the type strains for the closest related species were downloaded from EzBioCloud or GenBank, whereas the *rpoB* and *gyrB* gene sequences of the closest species were downloaded from GenBank. These three genes were individually aligned with PhyloSuite version 1.2.2 and the MAFFT version 7 plugin, concatenated with PhyloSuite and exported in FASTA format. A maximum likelihood phylogenetic tree was built using PhyloSuite and the IQ-TREE version 2.2.0 plugin. A GTR substitution model was used, according to Modelfinder plugin. FigTree version 1.4.4 was used to pre-edit the phylogenetic tree, and the final image was obtained in Inkscape version 1.0.1.

### 2.6. Genomic Taxonomy Analysis

To carry out the genomic taxonomy analysis, the whole-genome sequences of type strains ‘*Bacillus aerolatus*’ CX 253^T^ (WEIO01000001.1), *Bacillus badius* NBRC15713^T^ (NZ_BCVF01000001.1), *Bacillus thermotolerans* SGZ8^T^ (JWJE02000001.1), *Bacillus wudalianchiensis* FJAT27215^T^ (NZ_MAYT01000001.1) and *Bacillus xiapuensis* FJAT46582^T^ (NJAY01000001.1) were downloaded from the GenBank Database https://www.ncbi.nlm.nih.gov/genome (accessed on 6 September 2024). The Average Nucleotide Identity (ANI) was calculated according to the OrthoANI algorithm using the OrthoANI tool version 0.93.1 [23]. The DNA-DNA hybridization (DDH) value was determined in silico for these genomes using Genome-to-Genome Distance Calculator (GGDC) v.3.0 by the Basic Local Alignment Search Tool (BLAST) method. Genome-wide super tree building was performed by Type Strain Genome Server (TYGS) https://tygs.dsmz.de (accessed on 6 September 2024) [24]. The strain was also classified according to the Genome Taxonomy Database Toolkit (GTDB-tk) [25] using the Galaxy platform https://usegalaxy.org.au/ (accessed on 16 November 2024).

### 2.7. Genome Sequence Deposit

The whole-genome shotgun sequence of B190/17 was deposited in GenBank under the accession number JAUIYO000000000.

### 2.8. Addition of B190/17 Spectra to VITEK^®^ MS RUO and MALDI Biotyper^®^ Database

The SuperSpectrum of B190/17 was created and added to the Saramis Premium software version 4.0.0.14 database of VITEK^®^ MS RUO, as previously described by Costa et al. [1]. For addition to the MALDI Biotyper^®^ database, B190/17 was streaked on SBA and incubated at 30–35 °C for 24–48 h. Then, a portion of the colonies was suspended in 300 μL of sterile water, and after vigorous homogenization, 900 μL of absolute ethanol (Merck KGaA, Darmstadt, Germany) was added. After another vigorous homogenization, the sample was centrifugated at 13,000–15,000 rpm for 2 min. The supernatant was discarded, and centrifugation was performed again to ensure its complete removal. Then, the pellet was dried at room temperature for 3–5 min with the tube cap open. Next, 25–100 μL of 70% formic acid (Merck KGaA, Darmstadt, Germany) was added, and after homogenization, the same volume of acetonitrile (Merck KGaA, Darmstadt, Germany) was added to the pellet. After further centrifugation under the same conditions, one microlitre of supernatant was applied to eight spots of the slide, and one microlitre of BTS was applied to only one. After drying, one microlitre of Bruker HCCA was added to the spots. Calibration was performed in a BTS spot with Flex Control version 3.4, according to the manufacturer’s instructions. For spectrum acquisition, the eight sample spots were measured three times with Flex Control. The selection of the most homogeneous spectra of the sample and the elimination of the discrepant ones were carried out in Flex Analysis version 3.4, according to the manufacturer’s instructions, so that the main spectrum profile (MSP) could be created with at least 20 spectra. In MBP Compass Explorer version 4.1, the new MSP was compared with all the existing MSP libraries and then inserted into the customized library. Finally, the new version of this library was imported into the MBT Compass HT version 5.1.100. Afterwards, B190/17 was subjected once again to proteomic analysis by MALDI Biotyper^®^ in duplicate, as previously described, so that the new MSP could be challenged as to its suitability for identifying the desired strain. The results with score values < 1.70 were considered not identified; those between 1.70 and 1.99 were considered to have low confidence for species identification; and scores ≥ 2.00 were considered to have high confidence for species identification.

## 3. Results and Discussion

Costa et al. [1,8] previously isolated the strain B190/17 from an air monitoring sample at an immunobiological production facility and proposed that it could be designated as a new bacterial species. B190/17 was not identified by VITEK^®^ 2 [8] but was given the bionumber 0303101000000000 for its biochemical profile. It was only positive for the following biochemical tests: alanine arylamidase, Ala-Phe-Pro arylamidase, ELLMAN, leucine arylamidase, phenyalanine arylamidase and tyrosine arylamidase. Ellman’s reagent (5,5′-dithio-bis-(2-nitrobenzoic acid)—DTNB) is a colorimetric probe used to determine biothiols and proteins with free cysteine residues. The strain was negative for all the biochemical tests of API^®^ 50 CH and was not motile. The VITEK^®^ 2 biochemical test results are present in Table 1. A comparison of the phenotypic characteristics of B190/17 with reference strains is given in Table 2. Biochemical methods are not suitable for the reliable identification of *Bacillus* and related genus species.

Due to its limited and outdated database, the BCL card of VITEK^®^ 2 claims to identify 21 *Bacillus species* [4,7,28] (Table 3). However, currently, the number of identifiable *Bacillus* species is only seven since fourteen species are now classified in other genera, for example, *Bacillus megaterium* to *Priestia megaterium* and *Bacillus circulans* to *Niallia circulans* [11,13,28].

In the pharmaceutical industry, the accurate identification of microorganisms is very important, as a misidentification could lead to product release based on a false negative or product withdrawal due to a false positive result [5]. Furthermore, according to EU Annex 1, microorganisms detected in grade A and B areas (cleanrooms used for high-risk activities) should be identified to the species level, as well as spore-forming microorganisms isolated from grade C and grade D areas (cleanrooms used for low-risk activities), which should enable a robust risk assessment to reach and eliminate the source of contamination [3]. Therefore, there is a need to apply rapid, yet accurate, identification methods.

**Figure 1:**
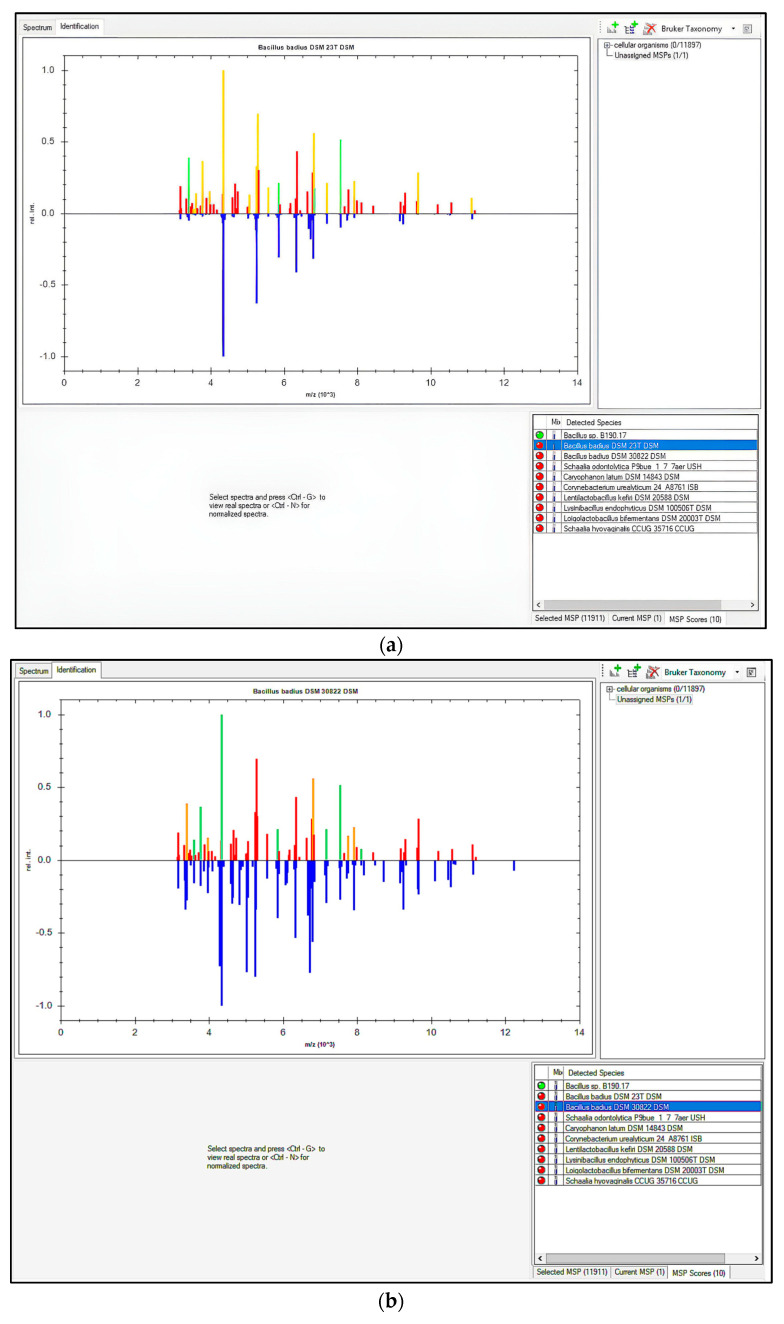
Comparison between spectra provided by MALDI Biotyper^®^ for B190/17 strain and *B. badius*. (**a**) Comparison between spectra of B190/17 and *B. badius* DSM 23^T^. (**b**) Comparison between spectra of B190/17 and *B. badius* DSM 30822. Ten closest matching classification results and their score values are displayed in box. Traffic-light colour-coding markers indicate closeness of match (green = full match; yellow = partial match; red = no match). Higher half of graphic displays peak list of B190/17, and its colour reflects degree of matching with reference main spectrum profile (MSP), whereas lower half displays peak list of selected MSP in blue using inverted scale.

MALDI-TOF MS is an appropriate methodology for use by microbiological control laboratories in the pharmaceutical industry. It is not laborious, and the results can be quickly obtained. However, its database needs to be regularly expanded and updated [1,2,4]. In this study, MALDI Biotyper^®^ and VITEK^®^ MS RUO from Bruker and bioMérieux were used as they are the only companies that sell MALDI-TOF MS in Brazil. At the time of this study, VITEK^®^ MS prime had not been launched. Neither of these MALDI-TOF MS systems were able to identify the B190/17 strain. Figure 1 shows a comparison between the spectra provided by MALDI Biotyper^®^ for the B190/17 strain and the two closest strains of the database: *B. badius* strains DSM 23^T^ and DSM 30822. It was apparent that the spectrum of the B190/17 strain matched just four and seven peaks, respectively, with *B. badius* DSM 23^T^ and *B. badius* DSM 30822. These matches were not enough for a reliable identification.

Most of the studies related to *Bacillus* species taxonomy are based on the 16S rRNA gene sequences [12]. If the 16S rRNA sequence of the target strain is compared to sequences in EzBioCloud or GenBank and there are no species with similarity >98.7%, then it is an indication that it may be a new species. Therefore, further genomic analysis is recommended [29]. Herein, 16S rRNA gene sequence analysis showed that all the *Bacillus* strains, including the strains without validly published nomenclatural status, and *Domibacillus* strains compared to the B190/17 strain showed similarity that was <98.7%. This supported the earlier proposal by Costa et al. [1,8] that B190/17 was a new bacterial species. By convention, B190/17 would be the type strain of the new species.

**Figure 2:**
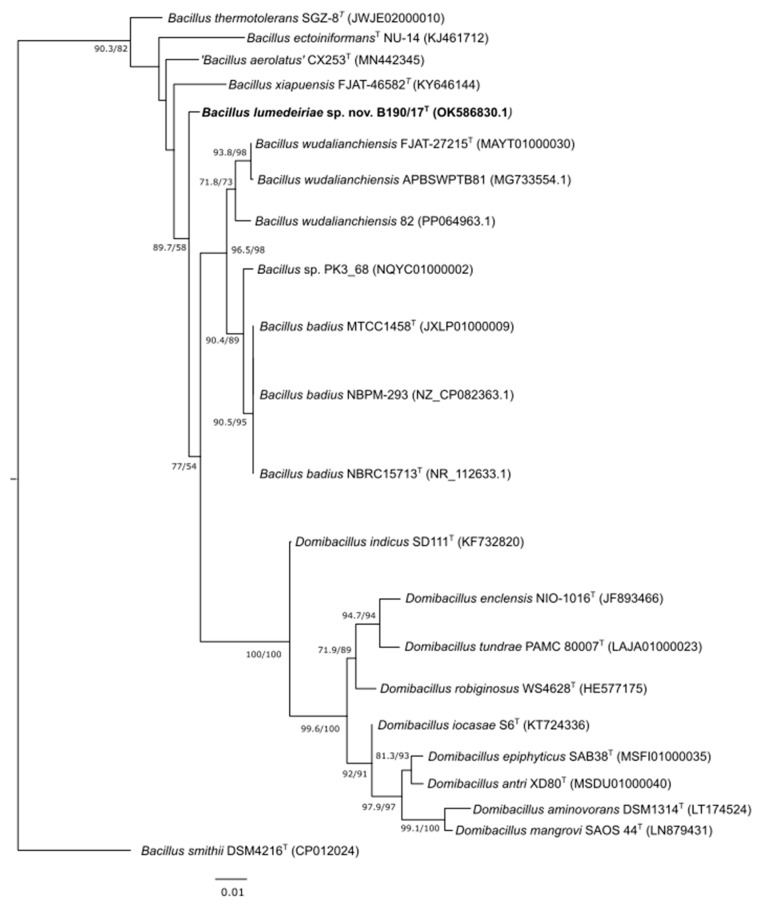
Maximum likelihood phylogenetic tree showing position of B190/17 strain based on 16S rRNA gene sequences (22 strains with 1487 bp). SH-arLT/bootstrap values (>50%) based on 5000 repetitions are shown. Sequence of *Bacillus smithii* DSM 4216 was used as outgroup. Bar 0.01% estimated sequence divergence. GenBank accession number is given, and type strains are indicated (T). SH-aLRT = Shimodaira–Hasegawa approximate likelihood ratio test.

**Figure 3:**
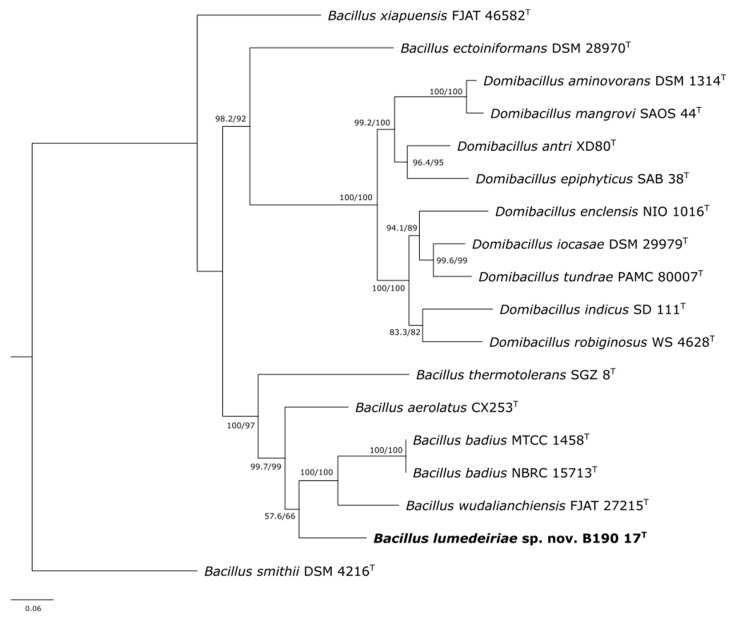
Maximum likelihood phylogenetic tree based on sequences of 16S rRNA, *rpoB* and *gyrB* showing position of B190/17 strain against most closely related strains (18 strains with 6973 nucleotides). SH-arLT/bootstrap values (>50%) based on 5000 repetitions are shown. Strain *Bacillus smithii* DSM 4216 was used as outgroup. Bar 0.06% estimated sequence divergence. Type strains are indicated (T). SH-aLRT = Shimodaira–Hasegawa approximate likelihood ratio test.

The phylogenetic tree based on 16S rRNA gene sequences revealed that the designated type strain B190/17 formed a separate branch closest to the *B. badius* MTCC 1458^T^ and *B. wudalianchiensis* FJAT 27215^T^ strains (Figure 2), whose similarities were 97.96 and 98.51%, respectively. *B. xiapuensis* FJAT 46582^T^, ‘*B. aerolatus*’ CX253^T^, *B. ectoiniformans* NU-14^T^ and *B. thermotolerans* SGZ8^T^ were placed in different branches and showed similarities of 97.63, 98.28, 96.49 and 97.21% with the 16S rRNA gene sequence from the B190/17 strain, respectively. All other type strains shared less than 96.49% 16S rRNA gene similarities in comparison with the B190/17 strain. ‘*Bacillus aerolatus*’ was described in 2020 by Chen et al. [26]. However, this species name has not been validated by the International Code of Nomenclature of Prokaryotes (ICNP). Although the species name is not validly published, its actual taxonomic status is referred to as ‘preferred name’ according to the List of Prokaryotic names with Standing in Nomenclature [13], and it has not been considered a synonym with any other species so far. Therefore, as the species ‘*Bacillus aerolatus*’ was one of the most closely related species to the B190/17 strain, it was decided to retain it in this study for comparative purposes to avoid the risk of not comparing a strain that could be the same species as B190/17. *Bacillus smithii* was used as an outgroup. This species was chosen because according to a maximum likelihood phylogenetic tree for 303 genome-sequenced *Bacillaceae* species (based on concatenated sequences for 650 core proteins), it was the closest species to the genera *Domibacillus* and *Pseudobacillus*, but it was in a distinct clade [11]. At the time of Gupta et al.’s [11] study, *B. badius* and *B. wudalianchensis* were classified as *Pseudobacillus badius* and *Pseudobacillus wudalianchensis* [30]. Thus, *Bacillus smithii* was used as an outgroup to enhance the resolution of the 16S rRNA gene phylogenetic trees (Figure 2 and Figure 3).

A phylogenetic analysis of *Bacillus* species has also been performed using other housekeeping genes [1,26]. However, there is not a standardized multilocus sequence analysis (MLSA) for the differentiation of this group of bacteria. The target genes are chosen according to each group of *Bacillus* species [9,31,32,33,34]. For the description of *Bacillus thermotolerans* [34] and *Bacillus aerolatus* [26], *gyrB* was analyzed in addition to 16S rRNA. Among the genes used to differentiate *Bacillus* species, *rpoB* and *gyrB* are the most commonly used [1,9,31,32,33,34]. Therefore, to confirm the 16S rRNA gene analysis, further analysis was carried out using the concatenated sequences of 16S rRNA (obtained by Sanger sequencing) *rpoB* and *gyrB* genes from the whole-genome sequences of the B190/17 strain. There is no cut-off percentage suggested in the literature for species and genus identification based on the analysis of the *rpoB* and *gyrB* genes as there is for the 16S rRNA gene. Therefore, they should be analyzed on a case-by-case basis by considering the phylogenetic analysis of the genes [1]. The concatenated phylogenetic tree also showed that the B190/17 strain was in a separate branch from ‘*B. aerolatus*’ CX253, *B. badius* MTCC 1458 and NBRC 15713 and *B. wudalianchiensis* FJAT 27215 type strains (Figure 3). The percentage of similarity of the *rpoB* and *gyrB* sequences of the B190/17 strain and their closest neighbours were ‘*B. aerolatus*’ CX253^T^ (87.50%, 85.43%), *B. badius* NBRC 15713^T^ (86.91%, 80.90%) and *B. wudalianchiensis* FJAT 27215^T^ (87.38%, 81.17%).

The genomic taxonomy results of B190/17 are shown in Table 4 and Table 5. The average size of the B190/17 genome was 3.43 Mb. The DNA G+C % content was 41.6 mol%, and the coverage was 73×. The assembly produced 89 contigs with a total of 3,434,160 bp, N50 of 219,177 bp and 3544 coding sequences. Other sequenced genome metrics provided by RAST are shown in Table 5. The 16S rRNA gene sequence obtained with Sanger sequencing (OK586830.1; 1499 bp) was also compared with the 16S sequence of the B190/17 genome (JAUIYO000000000; 1355 bp), and the percentage identity was 99.92%. Genome sequencing provided just one copy of the 16S sequence, and the de novo assembly approach was unable to recover all the copies of the 16S gene. Further, long-read sequencing was carried out to recover all the copies of the 16S gene and to close the genome. For Sanger sequencing, the MicroSEQ™ Full Gene 16S rDNA kit was used, which provided a 1499 bp sequence, larger than the 16S sequence of the genome, with very good quality.

The ANI values of the B190/17 strain and the related species ‘*B. aerolatus*’ CX253^T^, *B. badius* NBRC 15713^T^ and *B. wudalianchiensis* FJAT 27215^T^ were 79.55%, 76.47% and 77.64%, respectively, which are lower than the cut-off value (95–96%) established to consider as belonging to the same species [35]. Moreover, the estimations of in silico DNA–DNA hybridization (in silico DDH) by using the GGDC in comparison to the same type species above were 24.00, 21.60 and 22.50, respectively. All values are also lower than the cut-off value (70%) proposed for the delineation of novel species, indicating that they are distinct species [35].

**Figure 4:**
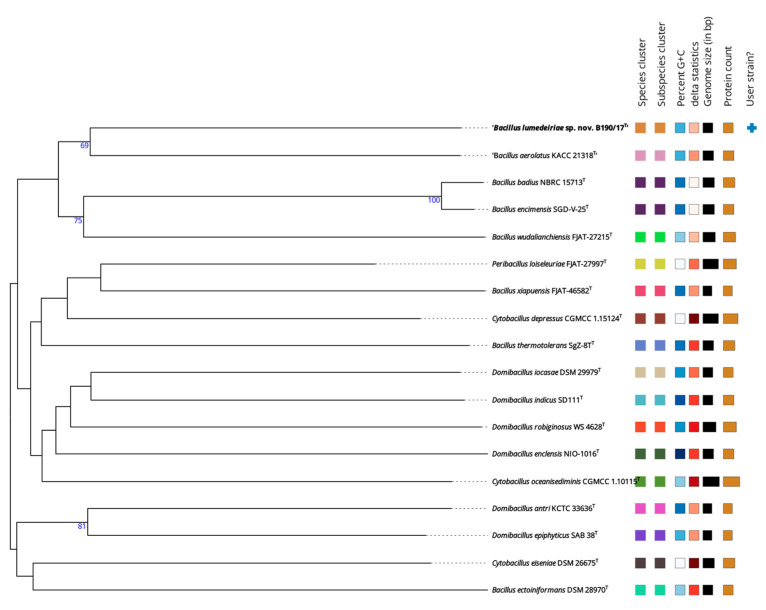
A phylotaxonomic genome tree showing the position of the B190/17 strain against the most closely related type strains based on whole-genome sequences. The genomic sequences were analyzed by type (strain) genome server (TYGS) with standard parameters. The tree was inferred with FastME 2.1.6.1 from GBDP distances calculated from genome sequences. The branch lengths are scaled in terms of GBDP distance formula d5. The numbers above branches are GBDP pseudo-bootstrap support values (>60%) from 100 replications, with an average branch support of 36.3%. *Bacillus encimensis* is synonym of *Bacillus badius*, and *Bacillus aerolatus* is not validly published [13]. The colour of the species and subspecies cluster are based on dDDH (digital DNA-DNA hybridization). The minimum percentage G+C is represented by white squares and the maximum by dark blue. The width of the black square/rectangle indicates the genome size in bp. The width of the beige square/rectangle indicates the protein count. The user strain is flagged with a blue cross.

The phylotaxonomic tree constructed on the TYGS server provided further evidence for the distinct taxonomic status of the B190/17 strain within the genus *Bacillus*. Figure 4 shows the position of the B190/17 strain in comparison with the most closely related type strains based on whole-genome sequences. B190/17 is in a different branch from its closer relatives ‘*B. aerolatus*’, *B. badius* and *B. wuadalianchiensis*. These results supported the earlier conclusion that the B190/17 strain represents a novel species of the genus *Bacillus*. The new species is proposed as *Bacillus lumedeiriae* sp. nov., with the type strain B190/17^T^.

In the past, the criterion used by taxonomists to classify a *Bacillus* species was its ability to produce spores in the presence of oxygen. However, whole-genome studies of the *Bacillus* genus have resulted in the reclassification of many former *Bacillus* species into new genera. Gupta et al. [11] and Patel and Gupta [12] proposed that the *Bacillus* genus should be composed only of two clades and that strains not in these clades should be transferred to new genera. The two clades are the “Subtilis clade”, composed of *Bacillus* sensu stricto, and the “Cereus clade”, containing a variety of important pathogenic species. However, some species that do not form part of these two clades are still part of the genus *Bacillus*. These include the closest species to the B190/17 strain, *B. badius*, *B. thermotolerans*, *B. wudalianchiensis* and *B. xiapuensis* [13], despite the phylotaxonomic genome tree showing the distance among them. According to GTDB, the B190/17 strain was identified just at the genus level as *Pseudobacillus*, which corroborates the previous results that B190/17 is a new species and should comprise the same genus as *B. badius* and *B. wudalianchiensis*. Verma et al. [30] proposed the reclassification of *B. badius* and *B. wudalianchiensis* into the new genus *Pseudobacillus*, as mentioned before. The delineation of a new genus is much more complex than the delineation of a species, demanding the analysis of at least 30 genes in addition to phylogenomic comparisons [29,35]. Currently it remains for further studies to justify the reclassification of ‘*B. aerolatus*’, *B. badius*, *B. wudalianchiensis* and *Bacillus lumedeiriae* sp. nov. into a new genus.

Costa et al. [1] added the B190/17 strain in VITEK^®^ MS RUO simply as *Bacillus* spp. However, at that time, the genomic taxonomy analysis had not been concluded. Based on the conclusion that the B190/17 strain was a novel species of the genus *Bacillus*, it was added into the MALDI Biotyper^®^ database. The strain was again submitted to proteomic analysis and was identified as *Bacillus lumedeiriae* sp. nov. with a higher score = 2.32. Therefore, the addition of B190/17 to the MALDI Biotyper^®^ database will facilitate the identification of any further isolates.

According to 16S rRNA gene and whole-genome analysis, the closest valid species to *Bacillus lumedeiriae* sp. nov. are *B. badius*, *B. thermotolerans*, *B. wudalianchiensis* and *B. xiapuensis*. With the exception of *B. badius*, these species were not present in the MALDI Biotyper^®^ database. The database represents only 32 of the 110 species already described [36]. This shows how the database can be further expanded to improve the identification of *Bacillus* and related genera in microbiological control laboratories. Consequently, MALDI-TOF MS analysis can be used to ensure greater safety in the release of pharmaceutical products and to trace the sources of contamination within a pharmaceutical facility.

### Description of Bacillus lumedeiriae sp. nov.

*Bacillus lumedeiriae* (lu.me.dei.riae. N.L. gen. fem. n. medeiros) of Luciane Martins Medeiros was used in memoriam of this Brazilian scientist at the Institute of Immunobiological Technology (Bio-Manguinhos) of Fiocruz in Rio de Janeiro who made significant contributions to the Laboratory Microbiological Control of Bio-Manguinhos, including introducing new microorganism identification systems.

The cells are Gram-positive, spore-forming and non-motile and grow in aerobic conditions (24–48 h) at 30–37 °C (optimum temperature: 37 °C). Cream, non-mucoid, circular, irregular edge, smooth, brilliant and medium colonies (up to 6 mm in diameter) were observed after 24 h of incubation on TSA, at 37 °C. The type strain was found to be positive for alanine arylamidase, Ala-Phe-Pro arylamidase, cysteine residues, leucine arylamidase, phenyalanine arylamidase and tyrosine arylamidase. The activities of α- and β-glucosidase and β-manosidase were not detected. Regarding the acid production capacity, this was not observed for galactose, glycogen glucose, mannitol or ribose.

The genome size of B190/17 strain is estimated at 3.43 Mb with 41.6 mol% of G+C content. The genome was deposited in GenBank under access number JAUIYO000000000. The type strain is B190/17 (=CBAS 1225^T^ = CCBG 2064^T^), isolated in 2017 from air environmental monitoring in an immunobiological production facility in Brazil.

## Figures and Tables

**Table 1:** Biochemical profile (bionumber 0303101000000000) of B190/17 strain identified by VITEK^®^ 2.

Biochemical Test	Result	Biochemical Test	Result	Biochemical Test	Result	Biochemical Test	Result	Biochemical Test	Result	Biochemical Test	Result
BXYL	−	LysA	−	AspA	−	LeuA	+	PheA	+	ProA	−
BGAL	−	PyrA	−	AGAL	−	AlaA	+	TyrA	+	BNAG	−
APPA	+	CDEX	−	dGAL	−	GLYG	−	INO	−	MdG	−
ELLM	+	MdX	−	AMAN	−	MTE	−	GlyA	−	dMAN	−
dMNE	−	dMLZ	−	NAG	−	PLE	−	IRHA	−	BGLU	−
BMAN	−	PHC	−	PVATE	−	AGLU	−	dTAG	−	dTRE	−
INU	−	dGLU	−	dRIB	−	PSCNa	−	NaCI 6.5%	−	KAN	−
OLD	−	ESC	−	TTZ	−	POLYB_R	−				

BXYL = Beta-xylosidase; LysA = L-lysine arylamidase; AspA = L-aspartate arylamidase; LeuA = leucine arylamidase; PheA = Phenilalanine arylamidase; ProA = L-Proline arylamidase; BGAL = Beta galactosidase; PyrA = L-Pyrrolidonyl arylamidase; AGAL = Alpha galactosidase; AlaA = alanine arylamidase; TyrA = tyrosine arylamidase; BNAG = Beta-N-acetyl-glucosaminidase; APPA = Ala-Phe-Pro arylamidase; CDEX = Cyclodextrine; dGAL = D-galactose; GLYG = glycogen; INO = Myo-inositol; MdG = Acidification methyl-A-D-glucopiranoside; ELLM = Ellman; MdX = Methyl-D-xyloside; AMAN = alpha-mannosidase; MTE = Maltotriosis; GlyA = Glycine arilamidase; dMAN = D-mannitol; dMNE = D-mannose; dMLZ = D-melezitosis; NAG = N-acetyl-D-glucosamine; PLE = Palatinose; IRHA = Ramnose; BGLU = Beta-glucosidase; BMAN = Beta-manosidase; PHC = Phosphoryl choline; PVATE = pyruvate; AGLU = Alpha-glucosidase; dTAG = D-tagatose; dTRE = D-trealose; INU = Inulina; dGLU = D-glucose; dRIB = D-ribose; PSCNa = Putrescine assimilation; NaCl 6.5% = growth in NaCl 6.5%; KAN = Kanamycin resistance; OLD = Oleandomycin resistance; ESC = Sulin hydrolysis; TTZ = Tetrazolium red; POLYB_R = Polymyxin_B resistance; ‘−’ = 0% to 5% positive; ‘+’ = 95 to 100% positive.

**Table 2:** Phenotypic characteristics of strain B190/17 and reference strains.

Characteristics	1	2	3	4	5	6
Cell shape	rod	rod	rod	ovoid	rod	rod
Motility	−	+	+	−	+	+
Optimum temperature for growth (°C)	37	37	30	50	37	25
Catalase	+	+	+	+	+	+
Starch	−	−	−	−	−	−
L-arabinose	−	−	−	−	−	−
L-rhamnose	−	−	−	−	−	ND
Lactose	−	−	−	−	−	−
Glucose	−	+	−	−	−	+
L-sorbose	−	−	−	+	−	ND
Mannitol	−	+	−	−	−	−
Sucrose	−	+	−	−	−	−
Amygdalin	−	+	−	−	−	ND
Inositol	−	+	−	−	−	−
G+C content (%)	41.6	42.3	41.2	44.4	44.0	44.2

Strain: 1, *Bacillus lumedeiriae* sp. nov. B190/17^T^; 2, ‘*B. aerolatus*’ CX253^T^; 3, *B. wudalianchiensis* CCTCC AB 2015266^T^; 4, *B. thermotolerans* CCTCC AB 2012108^T^; 5, *B. badius* JCM 12228^T^; 6, *B. xiapuensis* FJAT-46582^T^. Five strains were oxidase-negative and do not hydrolyse casein, sodium thiosulfate, tryptophan or pyruvate. +, Positive; −, negative. ND, not described. Data for B190/17 were taken from this study. Data for strains 2–5 were taken from Chen et al., 2020 [26], and for strain 6, they were taken from Liu et al., 2019 [27].

**Table 3:** A comparison between the number of *Bacillus* and related genus species described in the literature (Parte et al., 2020 [13]) (accessed on 8 September 2024) and the species present in the VITEK^®^ 2 database for the BCL card (bioMérieux, 2019 [28]).

Genera	Number of Species/Group of Species in VITEK^®^ 2 Database	Number of Species Described
*Alicyclobacillus*	1	29
*Aneurinibacillus*	1	9
*Bacillus*	21	111
*Brevibacillus*	8	33
*Geobacillus*	4	12
*Lysinibacillus*	1	22
*Paenibacillus*	14	310
*Virgibacillus*	2	34

**Table 4:** Genomic taxonomy results of B190/17 strain compared to five closely related taxa.

Strains	16S rRNA (%)	*rpoB* (%)	*gyrB* (%)	Ortho ANI (%)	GGDC (%)	Mol GC Distance (%)
‘*Bacillus aerolatus*’ CX253^T^	98.28	87.50	85.43	80.01	24.00	0.70
*Bacillus badius* NBRC 15713^T^	97.96	86.91	80.90	76.97	21.60	2.33
*Bacillus thermotolerans* SGZ8^T^	97.21	84.09	NSSF	73.73	20.10	2.81
*Bacillus wudalianchiensis* FJAT 27215^T^	98.51	87.38	81.17	78.22	22.50	0.39
*Bacillus xiapuensis* FJAT 46582^T^	97.63	82.91	NSSF	72.82	21.10	2.63

ANI, Average Nucleotide Identity; AAI, Average Amino Acid Identity; GGDC, Genome-to-genome Distance Calculator. ‘Species not validly published’, according to the List of Procaryotic names with Standing in Nomenclature (Parte et al., 2020) [13]. T, type strain; NSSF, no significant similarity found.

**Table 5:** General features of genome sequences of *Bacillus lumedeiriae* sp. nov.

Characteristics	B190/17
Estimated genome size (bp)	3,434,160
Coverage	73×
G+C content (%)	41.6
N50	219,177
L50	4
Number of contigs	89
Number of coding sequences	3544
Number of RNA genes	159

bp, base pair; RNA, ribonucleic acid.

## Data Availability

The original contributions presented in the study are included in the article, further inquiries can be directed to the corresponding author.

## References

[1] Costa L.V.D., Miranda R.V.D.S.L., Reis C.M.F., Andrade J.M., Cruz F.V., Frazão A.M., Fonseca E.L., Ramos J.N., Brandão M.L.L., Vieira V.V. (2022). MALDI-TOF MS database expansion for identification of *Bacillus* and related genera isolated from a pharmaceutical facility. J. Microbiol. Methods.

[2] Song M., Li Q., Liu C., Wang P., Qin F., Zhang L., Fan Y., Shao H., Chen G., Yang M. (2024). A comprehensive technology strategy for microbial identification and contamination investigation in the sterile drug manufacturing facility—A case study. Front. Microbiol..

[3] European Medicines Agency (2022). European Union guidelines for good manufacturing practice for medicinal products for human and veterinary use. The Rules Governing Medicinal Products in the European Union.

[4] Miranda R.V.D.S.L., da Costa L.V., Albuquerque L.S., Dos Reis C.M.F., Braga L.M.P.D.S., de Andrade J.M., Ramos J.N., Mattoso J.M.V., Forsythe S.J., Brandão M.L.L. (2023). Identification of *Sutcliffiella horikoshii* strains in an immunobiological pharmaceutical industry facility. Lett. Appl. Microbiol..

[5] Mattoso J.M.V., Costa L.V.C., Vale B.A., Reis C.M.F., Andrade J.M., Braga L.M.P.S., Conceição G.M.S., Costa P.B.M., Silva I.B., Rodrigues L.A.P. (2024). Quantitative and qualitative evaluation of microorganism profile identified in bioburden analysis in a biopharmaceutical facility in Brazil: Criteria for classification and management of results. PDA J. Pharm. Sci. Technol..

[6] Stamatoski B., Ilievska M., Babunovska H., Sekulovski N., Panov S. (2020). Optimized genotyping method for identification of bacterial contaminants in pharmaceutical industry. Acta Pharm..

[7] Caldeira N.G.S., de Souza M.L.S., de Miranda R.V.D.S.L., da Costa L.V., Forsythe S.J., Zahner V., Brandão M.L.L. (2024). Characterization by MALDI-TOF MS and 16S rRNA gene sequencing of aerobic endospore-forming bacteria isolated from pharmaceutical facility in Rio de Janeiro, Brazil. Microorganisms.

[8] Costa L.V.D., Miranda R.V.D.S.L., Fonseca E.L., Gonçalves N.P., Reis C.M.F., Frazão A.M., Cruz F.V., Brandão M.L.L., Ramos J.N., Vieira V.V. (2022). Assessment of VITEK^®^ 2, MALDI-TOF MS and full gene 16S rRNA sequencing for aerobic endospore-forming bacteria isolated from a pharmaceutical facility. J. Microbiol. Methods.

[9] Husni A.A.A., Ismail S.I., Jaafar N.M., Zulperi D. (2021). Current classification of the *Bacillus pumilus* group species, the rubber-pathogenic bacteria causing trunk bulges disease in Malaysia as assessed by MLSA and multi rep-PCR approaches. Plant Pathol. J..

[10] Qi H.Y., Wang D., Han D., Song J., Ali M., Dai X., Zhang X., Chen J. (2023). Unlocking antagonistic potential of Bacillus amyloliquefaciens KRS005 to control gray mold. Front. Microbiol..

[11] Gupta R.S., Patel S., Saini N., Chen S. (2020). Robust demarcation of 17 distinct *Bacillus* species clades, proposed as novel *Bacillaceae* genera, by phylogenomics and comparative genomic analyses: Description of *Robertmurraya kyonggiensis* sp. nov. and proposal for an emended genus Bacillus limiting it only to the members of the Subtilis and Cereus clades of species. Int. J. Syst. Evol. Microbiol..

[12] Patel S., Gupta R.S. (2020). A phylogenomic and comparative genomic framework for resolving the polyphyly of the genus *Bacillus*: Proposal for six new genera of *Bacillus* species, *Peribacillus* gen. nov., *Cytobacillus* gen. nov., *Mesobacillus* gen. nov., *Neobacillus* gen. nov., *Metabacillus* gen. nov. and Alkalihalobacillus gen. nov. Int. J. Syst. Evol. Microbiol..

[13] Parte A.C., Sardà Carbasse J., Meier-Kolthoff J.P., Reimer L.C., Göker M. (2020). List of Prokaryotic names with Standing in Nomenclature (LPSN) moves to the DSMZ. Int. J. Syst. Evol. Microbiol..

[14] Yoon S.H., Ha S.M., Kwon S., Lim J., Kim Y., Seo H., Chun J. (2017). Introducing EzBioCloud: A taxonomically united database of 16S rRNA gene sequences and whole-genome assemblies. Int. J. Syst. Evol. Microbiol..

[15] Woo P.C.Y., Lau S.K.P., Teng J.L.L., Tse H., Yuen K.-Y. (2008). Then and now: Use of 16S rDNA gene sequencing for bacterial identification and discovery of novel bacteria in clinical microbiology laboratories. Clin. Microbiol. Infect..

[16] Xiang C.-Y., Gao F., Jakovlic I., Lei H.-P., Hu Y., Zhang H., Zou H., Wang G.-T., Zhang D. (2023). Using PhyloSuite for molecular phylogeny and tree-based analyses. iMeta.

[17] Zhang D., Gao F., Jakovlić I., Zou H., Zhang J., Li W.X., Wang G.T. (2020). PhyloSuite: An integrated and scalable desktop platform for streamlined molecular sequence data management and evolutionary phylogenetics studies. Mol. Ecol. Res..

[18] Katoh K., Standley D.M. (2013). MAFFT multiple sequence alignment software version 7: Improvements in performance and usability. Mol. Biol. Evol..

[19] Kalyaanamoorthy S., Minh B.Q., Wong T.K.F., Von Haeseler A., Jermiin L.S. (2017). ModelFinder: Fast model selection for accurate phylogenetic estimates. Nat. Methods.

[20] Nguyen L.T., Schmidt H.A., von Haeseler A., Minh B.Q. (2015). IQ-TREE: A fast and effective stochastic algorithm for estimating maximum-likelihood phylogenies. Mol. Biol. Evol..

[21] Prjibelski A., Antipov D., Meleshko D., Lapidus A., Korobeynikov A. (2020). Using SPAdes De Novo Assembler. Curr. Protoc. Bioinform..

[22] Aziz R.K., Bartels D., Best A.A., DeJongh M., Disz T., Edwards R.A., Formsma K., Gerdes S., Glass E.M., Kubal M. (2008). The RAST Server: Rapid annotations using subsystems technology. BMC Genom..

[23] Lee I., Kim Y.O., Park S.C., Chun J. (2016). OrthoANI: An improved algorithm and software for calculating average nucleotide identity. Int. J. Syst. Evol. Microbiol..

[24] Meier-Kolthoff J.P., Göker M. (2019). TYGS is an automated high-throughput platform for state-of-the-art genome-based taxonomy. Nat. Commun..

[25] Chaumeil P.A., Mussig A.J., Hugenholtz P., Parks D.H. (2020). GTDB-Tk: A toolkit to classify genomes with the Genome Taxonomy Database. Bioinformatics.

[26] Chen P., Wang D., Ren Q., Wu J., Jiang Y., Wu Z., Pan Y., Zhong Y., Guan Y., Chen K. (2020). *Bacillus aerolatus* sp. nov., a novel member of the genus *Bacillus*, isolated from bioaerosols in a school playground. Arch. Microbiol..

[27] Liu G.H., Liu B., Liu Q.Y., Wang J.P., Che J.M., Zhang H.F., Lan J.L., Sengonca C. (2019). *Bacillus xiapuensis* sp. nov., isolated from marine sediment. Int. J. Syst. Evol. Microbiol..

[28] bioMérieux (2019). Ref 21345 - VITEK® 2 BCL. 045519- 02 - 2019-03, 1–24.

[29] Chun J., Oren A., Ventosa A., Christensen H., Arahal D.R., Costa M.S., Rooney A.P., Yi H., Xu X., Meyer S.D. (2018). Proposed minimal standards for the use of genome data for the taxonomy of prokaryotes. Int. J. Syst. Evol. Microbiol..

[30] Verma A., Pal Y., Ojha A.K., Kumari M., Khatri I., Rameshkumar N., Schumann P., Dastager S.G., Mayilraj S., Subramanian S. (2019). Taxonomic insights into the phylogeny of *Bacillus badius* and proposal for its reclassification to the genus *Pseudobacillus* as *Pseudobacillus badius* comb. nov. and reclassification of *Bacillus wudalianchiensis* Liu et al., 2017 as *Pseudobacillus wudalianchiensis* comb. nov. Syst. Appl. Microbiol..

[31] Dhruw C., Husain K., Kumar V., Sonawane V.C. (2020). Novel xylanase producing *Bacillus* strain X2: Molecular phylogenetic analysis and its application for production of xylooligosaccharides. 3 Biotech.

[32] Ben Gharsa H., Bouri M., Mougou Hamdane A., Schuster C., Leclerque A., Rhouma A. (2021). *Bacillus velezensis* strain MBY2, a potential agent for the management of crown gall disease. PLoS ONE.

[33] Cuellar-Gaviria T.Z., García-Botero C., Ju K.S., Villegas-Escobar V. (2023). The genome of *Bacillus tequilensis* EA-CB0015 sheds light into its epiphytic lifestyle and potential as a biocontrol agent. Front. Microbiol..

[34] Yang G., Zhou X., Zhou S., Yang D., Wang Y., Wang D. (2013). *Bacillus thermotolerans* sp. nov., a thermophilic bacterium capable of reducing humus. IJSEM.

[35] Riesco R., Trujillo M.E. (2024). Update on the proposed minimal standards for the use of genome data for the taxonomy of prokaryotes. Int. J. Syst. Evol. Microbiol..

[36] Bruker (2022). Ref 1854229 - Species/Entry List MBT Compass Library Revision K. Document Revision E. 1–75.

